# The Feasibility of a Land Ferry System to Reduce Highway Maintenance Cost and Associated Externalities

**DOI:** 10.1155/2016/8180232

**Published:** 2016-06-23

**Authors:** Steve J. Merrill, Alexander Paz, Victor Molano, Pramen P. Shrestha, Pankaj Maheshwari, Haroon Stephen, Hanns de la Fuente-Mella

**Affiliations:** ^1^Nevada Department of Transportation, 1263 South Stewart Street, Carson City, NV 89712, USA; ^2^Department of Civil and Environmental Engineering and Construction, University of Nevada, 4505 S. Maryland Parkway, P.O. Box 454015, Las Vegas, NV 89154-4015, USA; ^3^Facultad de Ciencias Económicas y Administrativas, Pontificia Universidad Católica de Valparaíso, Avenida Brasil 2830, 2340031 Valparaíso, Chile

## Abstract

This study provides an economic evaluation for a Land Ferry, which is a rail system capable of carrying trucks and all other types of vehicles, passengers, and cargo. The Land Ferry system involves a sliding loading system to roll heavy loads onto a flatbed; as a result, loading and unloading of all vehicles and cargo could be accomplished simultaneously. The evaluation for this system included (1) the design of a new track alignment over which the Land Ferry system would run, (2) evaluation of various sources of power, (3) estimation of how many local jobs the Land Ferry would generate, and (4) a benefit-cost analysis. It was estimated that the Land Ferry would create over 45,788 temporary jobs in Nevada during the three-year construction period and 318 permanent jobs during operation. The majority of the benefits were attributed to savings in travel time ($356.4 M), vehicle operating costs ($1000.4 M), reduction of accidents ($544.6 M), and pavement maintenance ($503.2 M). These benefits would be a consequence of the shift of trucks from the highway, thus resulting in higher speeds, decrease fuel consumption, and decrease vehicle maintenance costs. The overall benefit-cost ratio of 1.7 implies a cost-effective project.

## 1. Introduction

The transportation system in the United States experiences heavy freight traffic on both highways and railroads. Assuming that the number of trucks will continue to increase, it is very important that issues regarding maintenance, reliability, safety, and emissions be addressed. Alternatives to addressing these issues include

1. highway maintenance as is done currently (the “status quo” option),
2. adding more lanes,
3. adding more sidings to the existing railroads,
4. building additional rail systems and tracks,
5. building a rail system capable of carrying heavy trucks, that is, a Land Ferry.

The difference between the last two alternatives is the capacity and the related infrastructure needed to load and unload trucks instead of a container on a rail carriage. The Land Ferry alternative supports the transport of complete tractor-trailer rigs, with or without the driver. This alternative can offer the convenience of door-to-door service of trucks combined with cost savings associated with the long-haul economics of rail by utilizing a loading system to roll heavy trucks onto a flat bed. Complete trains can be loaded and unloaded quickly using side mechanisms; in other words, loading and unloading of all the trucks and vehicles can be accomplished simultaneously.

In this study, a simulation of the proposed concept was created to better illustrate these ideas [1]. Earlier implementations of this proposed system already are operating in Europe [2, 3]. The Land Ferry system represents a new multimodal alternative that partially removes trucks from the highway while maintaining the same flexibility currently provided when using trucks. Various countries use these multimodal locomotive systems to alleviate maintenance, reliability, and safety concerns.

One such system in India is the Roll-on Roll-off (RoRo) system. Studies conducted on the most comparable routes, approximately 400 miles long, showed that when the RoRo system operated at full capacity (40 trucks), the total fuel savings were around 10 liters of fuel per kilometer, or 1,407 gallons of fuel per trip [4]. Moreover, the RoRo system has been proposed for water links in the US. They are popular among freight companies due to their flexibility and ability to integrate with other transport systems as well as their operational speed [5].

The CargoBeamer [6, 7], which is in operation across Europe, is perhaps the most similar system to the proposed Land Ferry. The CargoBeamer enables quick and quiet parallel unloading and loading of trains at terminals. Superstructures enable noncraneable semitrailers to be transferred from the road to the railcars delays in a very efficient manner.

The newest development is the carless train, such as the RoadRailer® and the RailRunner®, as well as the Iron Highway, which involves a vehicle that can travel easily on both by rail and on roadways [8, 9]. This technology saves weight and results in reduced fuel costs, ultimately leading to savings that are passed onto the customer in terms of lower shipping costs. The major hindrances to this system include objections by organized labor and the weight limitations of roads.

In collaboration with the Federal Highway Administration (FHWA), studies have been conducted in the US to evaluate an intermodal rail-truck transport system for the western corridor of Interstate Highway 80 (I-80) [10–12]. This method reduces cargo handling and reduces damage and loss; furthermore, it allows freight to be transported faster than highways. Intermodal rail provides benefits for longer distances in terms of reduced costs as well as reduced greenhouse gas emissions [13–15]. For shorter distances, either trucking or the proposed Land Ferry is a better option in terms of travel time.

To evaluate the potential of a Land Ferry system, several factors needed to be considered. As a case study, the I-80 corridor on northern Nevada, which currently deals with heavy truck traffic and the associated high maintenance costs [16], was chosen to conduct a feasibility analysis. The corridor provided various opportunities to utilize renewable energies to power the Land Ferry system. This study took into consideration the location of the alignment, the costs for right-of-way and land acquisition, environmental costs, construction costs, maintenance and operation (O&M) costs, and user costs. In addition, economic impacts of the proposed Land Ferry system were estimated.

The area of interest for this research was the 320 mi stretch of the I-80 corridor connecting the towns of Fernley and Wells in northern Nevada. The location for the origin and destination were suggested by the Nevada Department of Transportation (NDOT), based on their experience and knowledge of the corridor. Other data used in this research were obtained from the National Map of the US Geological Survey [17].

The proposed Land Ferry system in northern Nevada would run for the most part parallel to a Union Pacific Railroad (UPR) line that has freight and Amtrak service. Discussions with UPR authorities suggested that the existing railroad currently was operating close to capacity due to the maintenance requirements of the track and bridges, limitations during flooding, at-grade crossings, and the need to share tracks between transport for freight and passengers. If the existing railroad was used to transport trucks, the system would reach capacity in the near future. This is especially true when considering the rise in freight traffic for such bulk commodities as coal, mineral ores, and chemicals moving to and from Nevada as well as for any other future developments. For example, the planned Tesla Lithium Ion factory in northern Nevada near Reno, the latest discovery of a large new source of gold in Elko, Nevada, and various warehousing activities recently added in the region are expected to increase the overall demand significantly along the corridor. To accommodate this massive rise, the existing railroad has to increase the capacity substantially; moreover, it is expected that upgrading the lines will cost millions.

The concept of the Land Ferry can be showcased to the freight companies operating in the region, demonstrating that the toll cost would be much lower than the per mile cost of roadway operations. Considering that similar concepts have been proven and tested in Europe and Asia, marketing should not be an issue as long as appropriate case studies and successful implementations are provided to the users.

This paper is organized as follows.  Section 2 discusses the methodology used to conduct this study.  Section 3 provides the results of the economic analysis and the benefit-cost analysis (BCA). Conclusions are presented in Section 4.

## 2. Methodology

The methodology used in this study consisted of six steps. First, the alignment of the proposed Land Ferry system was set up by using the available topographical maps. Second, the entire system of the Land Ferry was determined, including stations, loading platforms, loading boards, railcars and hydraulic systems, and loading and unloading systems. Third, based on the economic analysis, a power generation system was recommended to operate the Land Ferry. The capital cost estimation of the Land Ferry system was performed in this step. The fifth step consisted of an economic impact analysis to determine the economic output and permanent job creation in Nevada. The final step consisted of a benefit-cost analysis (BCA) to determine the benefits from the construction of this system.

### 2.1. Alignment

Taking into consideration the location of towns, local mines, the characteristics of the terrain, and the location of adjacent facilities, a procedure was developed to select the alignment for the Land Ferry. Six new stations were recommended in order to maximize the economic benefit: five full stations and a half station. Building six stations would allow trucks to serve the interior towns of northern Nevada, and the half station would serve the extensive mining interests in Nevada. The proposed alignment was selected by evaluating topography, socioeconomic factors, and environmental effects. In addition, the proposed alignment satisfied various design and operational constraints. Finally, it was important to provide an efficient route in terms of time and money [18, 19].

A methodology was developed to determine the optimal alignment that satisfied various design and operation constraints, such as gradient, length, curvature, construction, and operating and maintenance (O&M) costs. The following factors were considered to select the alignment:

1. Topography, using digital elevation models (DEMs).
2. Land cover, such as water bodies, wetlands, farmland, developed area, and forests.
3. Flood zones.
4. Landslides.
5. Optimum cut and fill volumes.

GIS is a tool for spatial analysis that has been used in transportation [20, 21]. In railway transportation, GIS has been used for alignment [22] and asset management [23]. The optimal alignment of a railway track requires finding the shortest path while satisfying various constraints originating from policies, regulations, and available resources [24–26]. We use DEM and land-cover information, among various other maps, to determine an optimal alignment with the help of GIS analysis. These maps were used to develop cells of a given dimension, which were assigned individual information or data [27]. It was beneficial to overlay this data using weights to assign values to each cell. The overlaid cells were used as cost factors for shortest-path algorithms. A raster calculator, a tool within the Spatial Analyst extension of ArcGIS, was used to convert the raster cells to the desired parameters. The optimal path, found using the ArcGIS raster and spatial analysis tools with weights and a set of parameters, was used afterwards to calculate the earthworks.

The next step was to use the tool “feature vertices to points” to generate points for all the vertices of the alignment. The “interpolate shape” tool from the 3D analyst was used to interpolate elevation values for these points along the alignment. These elevation values were taken from the DEM raster of the terrain, and the coordinates in *X*, *Y*, and *Z* were calculated for all points. Then, an optimization procedure was used to determine the optimal elevation that would minimize earthworks. The objective function was to minimize the absolute value of the difference in elevation between the proposed alignment and the actual terrain. By minimizing this difference, cut and fill volumes were balanced. This difference was evaluated every 1000 ft. A sensitivity analysis with multiple segment lengths was performed to determine this length. Constraints for this optimization included the maximum and minimum grades allowed for rail systems [28]. The results from the optimization displayed the coordinates of the desired Land Ferry alignment, including *X*, *Y*, and the optimal *Z*. A new shape file was generated using the *X*, *Y*, and optimal *Z* coordinates; at that point, a new raster image was generated with the optimal altitude, using a 3D analyst tool, Inverse Distance Weight (IDW). IDW used interpolations based on weighted distances to create an approximate surface with the optimal altitude values. Next, a buffer of 20 ft on each side of the Land Ferry alignment was created. The tool “extract by mask” was used to create maps from the DEM and IDW maps. These two maps represented the “before” and “after” states of the project. Finally, the “cut fill” tool was used to calculate the earth works identifying the volume change between the proposed surface (after) and the terrain surface (before).

### 2.2. System Description

The ability to move trucks on and off a train in an efficient and safe manner is crucial for the Land Ferry. If the loading and/or unloading times are too high, leading to longer delivery times, the Land Ferry is not an option that will be used by trucking companies.

#### 2.2.1. Stations

The stations will provide the conditions required for loading and unloading by having a long, raised, and wide lane parallel to the rails. A lane on each side of the rails will be required to enable simultaneous loading and unloading. The lanes will include walking spaces on the outside of the station to allow workers to get from section to section easily. Ramps on each end of the lanes will be required. One of the ramps will be used to drive the vehicle up into the platform where the sliding mechanism is installed. The other ramp will be used to drive the vehicle off the platform.

#### 2.2.2. Loading Platforms

Each station will consist of a number of loading platforms located along the station lanes, each with the ability to load or unload a loading board carrying a vehicle onto or off a single railcar. The loading platform will include a section in which strong, low-friction casters are installed on the bottom of a steel-loading board; this board could be rolled from the platform to the railcar. Guide rails will ensure that the boards slide into and from the railcars properly. Hydraulic arms and motors will be used to slide or roll the boards with the vehicles on them. They could be located underneath the walkway part of the loading section.

#### 2.2.3. Loading Boards

**Figure 1:**
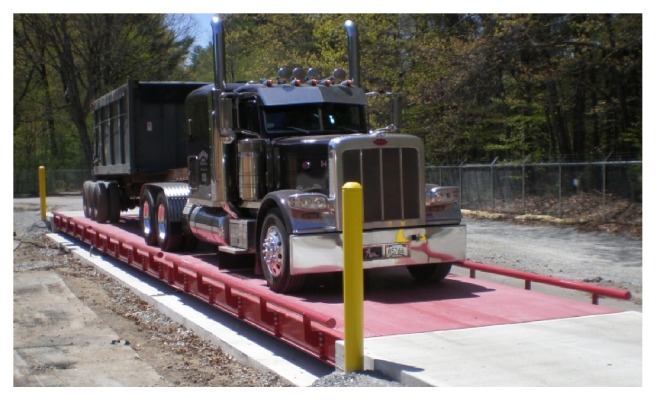
Scale with platform systems for the proposed Land Ferry [29].

The boards will be made of steel or similar weight-bearing material and have the strength required to support the weight of any vehicle(s), fully loaded. The boards will be as long as an average railcar and wide enough to fit a typical semitruck, with walking spaces on each side, similar to the scale in Figure 1. The boards will slide onto the railcars or platform sections, using hydraulic arms.

Guide rails will ensure that the boards slide into the railcars and platform sections properly. If loading boards already are in place on both sides of a platform, an auxiliary extra railcar could be used to relocate or remove one of the loading boards before the train arrives. Hence, a scenario involving the train going forwards or backwards will not occur.

Rows of railway trolleys will be required to support the weight of the loading board and a fully loaded semitrailer. Each loading board will have a series of durable, hard, rubber bumpers on each side to prevent them from bumping into each other. A wheel locking system will be used to tie down the vehicles in order to ensure that they do not move.

#### 2.2.4. The Railcar and the Hydraulics

Each railcar will be required to be equipped with a number of metal guide rails, which flare out at both ends. To line up the railcars precisely with the unloading spots, guide rails will be located on each side of the platforms. The board will be secured to the railcar by using a series of heavy-duty hooks. These hooks could be automated and will run along the length of the railcar on both sides.

The loading board is expected to be at a horizontal level and will be designed to support the total weight of the trucks. As the hydraulic cylinder lifts the loading board, it slides the loading board onto the railcar. The hydraulic cylinders need to be powerful enough to slide two boards, one on the platform section and one on the train. The first board slides from the platform onto the railcar. The second board slides from the railcar to the platform onto the other side of the rail.

#### 2.2.5. Loading/Unloading Process

Vehicles will approach the station, where they will use a ramp to go over the platforms to be positioned on their designated loading section. Then, the vehicles will be secured to a platform. As the train stops at the station, the hydraulic arms will slide the loading boards on the platform into contact with the boards on the train. The boards on the train will slide off into the platform section on the other side of the track. The boards on the train will be locked into position. The train and the vehicles that were unloaded then will be ready to leave the station.

With respect to drivers, when the truck is on the train, the driver will travel in a separate wagon. Another possibility will be to switch the drivers on the journey; that is, one driver will leave the truck at one end and another driver will pick up the truck at the other end. The logistics may be evaluated by the freight companies.

### 2.3. Power Generation

The amount of power required by the system depends on the tractive effort required by the Land Ferry. Tractive effort is a function of the total weight of the train and various characteristics along the alignment, such as grade [30, 31]. Usually, train resistance consists of curve, rolling, aerodynamic, wind, grade, and track resistance, as illustrated by

(1) $$\begin{array}{c} R=A+BV+CV^{2}, \end{array}$$

where *R* is rolling resistance, *V* is velocity, *A* is coefficient that varies with weight (“track” resistance), *B* is coefficient that varies directly with velocity, and *C* is coefficient that varies with the square of velocity (“aerodynamic” resistance).

An estimated 8 lb/ton is required to overcome rolling resistance for intermodal freight trains at a speed of 70 mph [32]. Curve resistance is approximately 0.8 lb/ton per degree of curvature for a standard gauge track [33]. It is assumed that a maximum degree of curve shall not exceed 5 degrees. Hence, curve resistance becomes 4 lb/ton. Grade resistance is the force required to overcome gradient and is equal to 20 lb/ton per 1% grade. Generally, a large amount of grade resistance is required when the train is moving uphill. The calculations assume a grade of 1.5% for the entire trip in order to provide conservative estimates, and grade resistance becomes 30 lb/ton. Overall, the total train resistance equals 42 lb/ton.

Amtrak's Acela Express and Northeast Regional trains have speed limits of 50 mph for freight trains on the existing corridor tracks [34]. The typical maximum speed of conventional freight trains in Europe ranges from 90 to 120 km/h (56 to 75 mph), and the predominant vehicle is a conventional freight wagon [35]. Considering that a new railroad track is being built for the Land Ferry, there is no impedance to the Land Ferry to accelerate and reach speeds up to 70 mph.

Horse power is calculated [28] by multiplying the tractive effort and the speed of the train and dividing by 375. For example, if the weight of the train is 1,200 tons (T), a tractive effort of 42 lb/T × 1,200 T = 50,400 pounds is required. The estimated horsepower (hp) of the train is 50,400 × 70/375 = 9,410 hp. In general, electric locomotives are approximately 85% efficient. Therefore, 9,410 hp is approximately equal to an electric motor that provides 9,410/0.85 = 11,070 hp = 11,070 × 746/1,000,000 = 8.25 MW. Usually for high-speed trains, if there are frequent stops and starts, the hp/ton ratio = 4. For the fast Union Pacific Southern (UPS) trains of the Burlington Northern Santa Fe Foundation (BNSF), hp/ton ratio = 6; for conventional passenger trains, hp/ton ratio = 4–8; and for rapid transit, hp/ton ratio = 10 [36]. Hence, it is possible to get a high hp/ton ratio to ensure rapid acceleration. In this case, the hp/ton ratio = 9.

This study evaluated various possibilities for the number of trips per day for each type of train size. The train will average approximately 70 mph over a distance of 320 miles. Therefore, the trip will take about 4.60 hours. Additionally, it is assumed that 12 minutes of wait time will be needed for the trucks to board the train and 12 minutes of wait time will be needed to exit the train. As a result, a total of 24 minutes (0.40 hours) was added to the transit time. For example, the electricity consumption for the train (30 trucks per train) to make the trip 25 times per day will equal 8.25 MW × 5 × 25 = 1031.25 MWh.

#### 2.3.1. Solar Photovoltaic

For northern Nevada, the average amount of sunlight in one day is approximately 5.5 to 6 hours [37]. The estimated size of a solar photovoltaic (PV) system is determined by dividing the MWh required by the average hours of direct sunlight. This equates to a system size of 1031.25 MWh/5.5 h = 187.5 MW. The average cost of a solar PV system would be approximately $2.00/watt [38]. The system would cost 187.5 × 1,000,000 × 2 = $375 M.

#### 2.3.2. Geothermal System

A geothermal plant can be used to produce electricity 24 hours a day. This means that the size of geothermal plant required for this system needs to be 1031.25 MWh/24 h = 42.97 MW. The average cost of a geothermal power plant ranges between $3 and $4 per watt [39]. The cost of a geothermal plant for this example would be 42.97 × 4 × 1,000,000 = $171.9 M.

#### 2.3.3. Wind Turbine System

Wind turbines of the most common utility scale have power capacities between 700 kW and 1.8 MW. The National Renewable Energy Laboratory (NREL) used a scale of 1 to 7, with 1 being “poor” and 7 being “superb,” to rate the areas in northern Nevada anywhere from 1 to 5 [40]. Wind farms can cost approximately $1,000/kW [41], and the cost of a wind farm would be 42.97 × 1000 × 1000 = $42.97 M.

#### 2.3.4. Diesel Engines

The fuel efficiency of a diesel locomotive is approximately equivalent to 450 mi/gal/T [42]. If the train weight is 1200 T, the corresponding mileage is 450/1200 = 0.38 mpg. The number of gallons consumed in the trip would be 320 mi/0.38 mpg = 842 gal. Assuming that the average cost of diesel fuel is $3.90/gallon, the cost per trip would be $3.90 × 842 = $3283.80. If the trip is made 25 times per day, the cost would become $3,283.80 × 25 = $82,095 or $82,095 × 365 = $30 M per year.

To evaluate the capital costs for each alternative, wind energy was the most economical, based on the analysis conducted in this study. However, to harness geothermal, wind, or solar energy, an electric power distribution system is required. The average capital costs of such installations are included in the construction costs for the Land Ferry. One important point is that the diesel costs are recurring; however, for geothermal, wind, or solar power, the costs are one time only.

### 2.4. Capital Cost Estimation

The methodology used in this paper for cost estimating was based on the historical bid and unit cost for typical designs. Due to the unavailability of both the final design and sufficient data, a standard typical design of railway tracks was used for estimation. Similarly, for station costs, a typical design of a station was used to calculate an estimated cost [43]. To estimate the detailed cost of the Land Ferry system, a work breakdown structure (WBS) was established. Every single item used to build the Land Ferry system was identified and placed into the WBS.

After the WBS was prepared, the cost of each work activity was estimated. The coding system allowed the estimated cost to be organized and presented by categories and subcategories. The cost-code categories and subcategories used in this estimate were taken from standard cost categories established by the Federal Railroad System (FRA) as part of American Recovery and Reinvestment Act (ARRA). Once the cost estimates for all categories were completed, they were compiled in a WBS table.

#### 2.4.1. Estimating Process

The historical lump-sum bid price was the method used when typical designs of the activities were not available. Unit cost analysis was used when a typical design was prepared and when the quantity takeoff of those activities was possible.

*(1) Historical Bid Price*. This study took into account the time the historical lump-sum bid price was prepared as well as the conditions of the historical project used for pricing. The following factors were applied as needed. The bid price of the items taken from other sources was adjusted from the current date by using appropriate escalation factors taken from* Engineering News Records (ENR)* [44]. In addition, the bid price was adjusted for location using location index from ENR. Historical lump-sum bid prices used in this estimate came from local, regional, statewide, and national high-speed rail projects.

*(2) Unit Cost Method*. For the proposed Land Ferry, the unit cost was used to estimate the cost of grading, cutting, filling, rock ballast, rails, ties, stations, and other minor items. This method allowed developing the unit cost based on the current local construction market. The production rate of the equipment and labor was taken from 2014 cost data from RS Means [45] to determine the equipment and labor time. The rental and operating costs of the equipment were taken from the rental book [46] of the California Department of Transportation (Caltrans) and adjusted for Nevada. The estimated labor cost was based on the percentage of the total cost provided in the RS Means cost guide. An overhead and profit cost of 20% was added to the direct cost of these items.

#### 2.4.2. Operations and Maintenance (O&M) Costs

The operating cost of the Land Ferry included the cost of power, oil, operators, and spare parts to operate the train. These costs were calculated based on number of trips and the distance travelled by using an historical unit price. In addition, the operating cost included the cost of running the stations, administrative buildings, maintaining the tracks and railroad engines, and other facilities and activities. These costs were estimated as 6% of the initial investment cost based on a 30-year life cycle. Research showed that, for a 30-year life cycle, the cost of buildings' utilities, services, and system replacement would be about 36%, the maintenance cost about 6%, and the initial cost of the project about 58% [47]. Other studies have shown similar results. For example, a life cycle cost study showed that, over the period of a 30-year life cycle, the operations and maintenance costs of the building are 6% of the total life cycle cost [48]. A study about green buildings showed that there was a significant correlation between the total construction cost and the maintenance cost [49]. The study also found that the maintenance cost of these buildings was about 15% of the initial cost of the project.

### 2.5. Economic Impact Analysis

The primary purpose of the Land Ferry is to reduce damage to roadways caused by consistent and intense heavy vehicle traffic, mainly commercial trucks used to ship goods along I-80 [13]. Aside from the physical benefits of keeping the trucks off the roads and keeping truckers from having to drive long, monotonous distances, the Land Ferry reduces truck maintenance, fuel costs, emissions, and accidents caused by tired driving [50]. A study conducted by the Virginia Tech Transportation Institute revealed that fatigue is a major factor in approximately 12% of all traffic accidents [51].

The purpose of this economic analysis was to determine the immediate and consequential effects to the regional economy. The Regional Input-Output Modelling System (RIMS II) was proposed to determine these effects based on finite, measurable changes to demand, earnings, and employment. Multipliers specific to each county were used in order to anticipate the direct, indirect, and induced effects. These multipliers were determined from nationwide, statewide, and countywide accounting tables, which were adjusted to reflect local conditions. The multipliers were applied directly to expected changes in the final demand (initial investment), earnings, and jobs. For the RIMS II model, 406 detailed industries were defined, each labeled using a specific six-digit industry code [52].

The multipliers chosen for this study resulted in three anticipated effects: output, earnings, and jobs. Output was equal to the sum of the direct investment, the indirect expenditures made after the first investment by households earning income directly from the investment, and the induced expenditures made by those receiving an income from these households. Output was determined by multiplying the initial investment by the final demand-output multiplier. Similarly, earnings were defined as the sum of direct, indirect, and induced incomes of all parties. This value was the product of the final demand change and the final demand earnings multiplier. The jobs generated were estimated as equal to the amount of jobs created for every $1,000,000 increase in the final demand change. Again, this value was equal to the sum of the direct, indirect, and induced job creation.

In this case, the employment ratios of Nevada were compared to those of the United States [53].  Table 1 shows all construction aspects that were considered, except for the contingency costs and contractors' profits. Therefore, it was assumed that the estimates were the minimum expected effects. This caused the final analysis cost to drop from $4,358,721,435 to $3,816,014,000.

### 2.6. Benefit-Cost Analysis

The final task of this study was a benefit-cost analysis of the Land Ferry for 40 years starting from 2016. For multibillion dollar projects, the benefits usually last much longer than the standard 30 years and must be fully reflected in the analysis. Considering the characteristics of the Land Ferry system as well as its long lasting effects, a 40-year horizon was more appropriate for this analysis [54]. Analysis of the Land Ferry system used the California Benefit-Cost (Cal-B/C) model [55], modified based on parameters for Nevada. This model estimated the effects of reduced trips on the existing I-80 and then placed those reduced trips on the Land Ferry to complete the analysis and account for all trips.

#### 2.6.1. Tolling Analysis

Regarding a tolling scheme for the proposed Land Ferry system, for the year 2012, it was assumed that an average of 30 trucks per train with 25 trips per day would generate 750 truck trips per day. The truck flows were assumed to increase at a rate of 2% per year [56]. According to latest report from the American Transportation Research Institute [57], the average total marginal cost for trucks in 2013 was $1.73/mile. Marginal costs were divided into vehicle-based and driver-based costs. According to this report, top costs for carriers were fuel/oil, driver wages, benefits, and truck/trailer lease or purchase payments.

The Land Ferry would run a length of 320 miles. As a result, the operational cost per truck per trip would be $1.73 × 320 = $554.00. The analysis showed that the annual expense in 2016 would be 812 × $554 × 365 = $164.04 M. Finally, the tolling revenue was estimated to be half the actual expenses because it would provide freight companies and drivers an incentive to use the Land Ferry.

#### 2.6.2. Crash Cost Savings

Based on the previous five-year crash data from the Nevada Citation and Accident Tracking System database of the Nevada Department of Transportation (NDOT), the study section averaged an annual of 12 fatal, 315 injury, and 760 property-damage-only (PDO) crashes. Of these, 70% were truck-related crashes. These crash statistics were specific to the study corridor on I-80 and did not include data for nearby or surrounding areas. Hence, the study section averaged an annual of nine fatal, 220 injury, and 532 PDO truck crashes.

In this analysis, 25% of the truck traffic would be removed from I-80 and transferred to the Land Ferry [54]. The savings would be in terms of three fatal, 55 injury, and 133 PDO crashes. Based on the crash costs provided by NDOT and adjusted for inflation, the estimated annual crash cost savings in year 2016 would be 3*∗*$5,900,000 + 55*∗*$165,000 + 133*∗*$10,600 = $28.2 M.

#### 2.6.3. Annual Pavement Rehabilitation Cost Savings

Shifting the trucks from the highway to the Land Ferry would directly reduce their effects on highway pavement and associated rehabilitation costs. The annual cost for routine roadway rehabilitation was based on actual costs incurred in similar rural regions throughout northern Nevada. Currently, the annual cost for pavement rehabilitation along the study corridor is about $10 million [58].

Once calculated, the cost per mile was applied to the reduction in rehabilitation costs on I-80. Studies have suggested that the daily maintenance cost due to a heavy truck (60 kip 5-axle comb/rural interstate) was $0.033/mile [59]. The base cost was assumed to increase by 2.5% annually due to inflation [60]. For 2016, the pavement rehabilitation cost would be $0.049/mile. Hence, the expected cost savings on annual pavement rehabilitation on the I-80 in 2016 were estimated as 812 × 0.049 × 320 × 365 = $4.65 M. Over 40 years, the total pavement rehabilitation cost savings would be $503.2 M. These are substantial savings in terms of expenditures on pavement maintenance.

## 3. Results

### 3.1. Results of the Economic Impact Analysis

Table 1 shows the results of the economic impact analysis. The initial $4.4 B investment in the Land Ferry could have a $6.1 B effect on the economic output of Nevada, $1.9 B worth of new income for Nevada residents, and 45,788 temporary jobs for Nevada locals during the construction phase. Over 40 years, the Land Ferry's O&M cost of $1.77 B ($44,209,000 × 40) would provide Nevada $1.83 B in output, $510 M in earnings, and 318 permanent jobs.

### 3.2. Results of the Benefit-Cost Analysis

Build and No-Build conditions were analyzed to model the benefits for the proposed system. In order to project growth in the corridor, trips on the Land Ferry were estimated using traffic information obtained from NDOT and travel demand models. This study used a 40-year horizon to estimate benefits and costs.

This system showed significant travel time savings ($356.4 M), vehicle operating cost savings ($1,000.4 M), and accident reductions ($544.6 M). This was expected due to the large percentage of trucks being removed from the I-80 corridor by the Land Ferry system. The costs and benefits for this system were approximately $4.36 B and $7.37 B, respectively. The benefit-cost ratio was 1.7, and the net present value benefit was $3.01 B.

### 3.3. Sensitivity Analysis

The benefit-cost analysis used a real discount rate of 7% [61]. A real discount rate is a discount rate that reflects the opportunity cost of money net of the rate of inflation [62]. A sensitivity analysis using a discount rate of 3% was recommended [63]. The costs changed from $4.36 B to $4.98 B, whereas the benefits changed from $7.37 B to $9.61 B. It was evident how sensitive the cost and benefits were, relative to a discount rate. Moreover, the benefit/cost ratio changed from 1.7 to 1.9, implying additional potential benefits.

## 4. Conclusions and Recommendations

This research provided an economic assessment of a Land Ferry system to reduce increasing costs of maintaining the I-80 transportation corridor in northern Nevada. The benefit-cost ratio for this project was 1.7 with a 7% discount rate. Hence, the system would provide significant benefits; consequently, it could be justified financially. Specifically, the benefits would be a consequence of improving travel by removing trucks from I-80 and transferring them onto a Land Ferry. The majority of the savings would be in terms of travel time, accident reductions, and vehicle operating cost. It should be noted that this analysis showed the cost effectiveness of an investment; in other words, the money invested would be returned to users and nonusers. It is not exactly a monetary return on investment.

From a funding perspective, a Public Private Partnership (PPP) model can be used to select a concessionaire that will build the Land Ferry facility and collect the tolling revenue. For example, a special rail expressway for freight, the Alameda Corridor, was opened in the US in 2002 to link the ports of Long Beach and Long Angeles to the big national rail routes. The corridor was completed on time and on budget of $2.4 billion by using a PPP model [34]. Another source of funding can be to attract private equity investors to finance, design, construct, and operate the facility as well as to provide tax breaks; this is similar to the funding sources recently approved for the car manufacturer, Tesla Motors, for a $5 B lithium-ion battery facility in northern Nevada.

The major contribution of this research is the comprehensive analysis of a multimodal system that has proven to be very effective internationally but has not yet been implemented in the US. The study illustrates all the tremendous potential of this type of system. In addition, this study provides detailed information on the GIS techniques used to identify the optimal alignment, description, and analysis of a loading and unloading mechanism for this system, analysis of various sources of power, monetary impacts on the local economy, and an overall benefit-cost analysis.

## Figures and Tables

**Table 1:** Results of the economic impact analysis.

	Item	Initial investment/final demand change	Total			Nevada		
			Output	Earnings	Jobs	Output	Earnings	Jobs
Phase one: construction								
1	Track	$1,902,389,000	$3,461,113,307	$965,644,351	22,604	$2,990,729,331	$860,582,489	19,912
2	Stations	$71,204,000	$135,692,390	$45,746,849	1,061	$119,325,898	$40,328,442	932
3	Support	$347,240,000	$677,986,100	$226,469,928	5,183	$673,885,850	$225,100,308	5,152
4	Land	$696,050,000	$1,300,811,425	$416,870,410	10,800	$1,297,056,768	$415,616,229	10,771
5	Communications	$79,150,000	$145,013,290	$39,807,930	1,074	$95,685,629	$25,564,444	664
6	Vehicles	$15,000,000	$24,108,300	$5,489,600	141	$16,839,258	$3,838,176	99
7	Professional services	$704,981,000	$1,303,199,892	$457,660,167	11,847	$908,719,582	$318,602,052	8,258

*Totals*		**$3,816,014,000**	$7,047,924,704	$2,157,689,235	52,709	**$6,102,242,316**	**$1,889,632,140**	**45,788**

*Proportion of total benefitting Nevada*						86.58%	87.58%	86.87%

Phase two: operations								
1	Utilities	$8,657,000	$15,984,285	$5,479,015	148	$15,984,285	$5,479,015	148
2	Fuel	$6,727,000	$11,662,600	$2,741,925	58	$11,662,600	$2,741,925	58
3	Operator	$328,000	$593,713	$141,729	3	$178,114	$42,519	1
4	Administrative maintenance	$1,443,000	$2,607,645	$865,511	39	$2,607,645	$865,511	39
5	Track maintenance	$26,304,000	$47,612,870	$11,365,958	224	$14,283,861	$3,409,788	67
6	Engine maintenance	$750,000	$1,357,575	$324,075	6	$923,151	$220,371	4

*Totals*		$44,209,000	$79,818,688	$20,918,213	478	**$45,639,656**	**$12,759,128**	**318**

*Proportion of total benefitting Nevada*						57.18%	61.00%	66.41%

*For 40 years*						**$1,825,586,234**	**$510,365,134**	**318**
